# Epidemiological exploration of the impact of bluetooth headset usage on thyroid nodules using Shapley additive explanations method

**DOI:** 10.1038/s41598-024-63653-0

**Published:** 2024-06-21

**Authors:** Nan Zhou, Wei Qin, Jia-Jin Zhang, Yun Wang, Jian-Sheng Wen, Yang Mooi Lim

**Affiliations:** 1 M. Kandiah Faculty of Medicine and Health Sciences, Universiti Tunku Abdul Rahman, Bandar Sungai Long, 43000 Kajang, Selangor Malaysia; 2 Department of Traditional Chinese Medicine and Modern Medicine, The First People’s Hospital of Nanning, No. 89, Qixing Road, Nanning, 530000 Guangxi Zhuang Autonomous Region People’s Republic of China; 3 Intervention Department, Dongtai City Traditional Chinese Medicine Hospital, No. 198 Yaju Road, High-Tech Zone, Dongtai, 224200 Jiangsu People’s Republic of China

**Keywords:** Thyroid nodules, Bluetooth headsets, Non-ionizing radiation, Shapley additive explanations, Lifestyle modification, Epidemiology, Risk factors

## Abstract

With an increasing prevalence of thyroid nodules globally, this study investigates the potential correlation between the use of Bluetooth headsets and the incidence of thyroid nodules, considering the cumulative effects of non-ionizing radiation (NIR) emitted by these devices. In this study, we analyzed 600 valid questionnaires from the WenJuanXing platform using Propensity Score Matching (PSM) and the XGBOOST model, supplemented by SHAP analysis, to assess the risk of thyroid nodules. PSM was utilized to balance baseline characteristic differences, thereby reducing bias. The XGBOOST model was then employed to predict risk factors, with model efficacy measured by the area under the Receiver Operating Characteristic (ROC) curve (AUC). SHAP analysis helped quantify and explain the impact of each feature on the prediction outcomes, identifying key risk factors. Initially, 600 valid questionnaires from the WenJuanXing platform underwent PSM processing, resulting in a matched dataset of 96 cases for modeling analysis. The AUC value of the XGBOOST model reached 0.95, demonstrating high accuracy in differentiating thyroid nodule risks. SHAP analysis revealed age and daily Bluetooth headset usage duration as the two most significant factors affecting thyroid nodule risk. Specifically, longer daily usage durations of Bluetooth headsets were strongly linked to an increased risk of developing thyroid nodules, as indicated by the SHAP analysis outcomes. Our study highlighted a significant impact relationship between prolonged Bluetooth headset use and increased thyroid nodule risk, emphasizing the importance of considering health impacts in the use of modern technology, especially for devices like Bluetooth headsets that are frequently used daily. Through precise model predictions and variable importance analysis, our research provides a scientific basis for the formulation of public health policies and personal health habit choices, suggesting that attention should be paid to the duration of Bluetooth headset use in daily life to reduce the potential risk of thyroid nodules. Future research should further investigate the biological mechanisms of this relationship and consider additional potential influencing factors to offer more comprehensive health guidance and preventive measures.

## Introduction

Thyroid nodules represent an abnormal growth within the thyroid gland, typically manifesting as localized swellings in thyroid tissue. The majority of these nodules are benign, asymptomatic, and do not necessitate intervention. Among cases diagnosed as malignant, they predominantly consist of small, slow-growing tumors, which can be safely managed with a conservative approach^1^. This study aims to explore the potential association between Bluetooth headset usage and the development of thyroid nodules. Given the possible effects of non-ionizing radiation on human health and the need for public awareness and preventive measures in technology usage, the motivation and objectives of this research hold substantial practical significance^2,3^. The prevalence of thyroid nodules in the global population is remarkably high, with a study from 2023 estimating an incidence rate of up to 67%^4^. This rate reflects a rapidly increasing trend, as corroborated by previous epidemiological data^5^. In China, a national epidemiological survey conducted in 2021 revealed a total prevalence of 36.9%^6^. Although thyroid nodules are predominantly benign, the potential risk of malignancy makes the increasing prevalence a concerning global health issue^7,8^. The increasing prevalence of thyroid nodules poses a significant challenge to healthcare professionals and policymakers worldwide. With globalization and technological advancements, understanding the epidemiological trends and potential risk factors of thyroid nodules has become increasingly important^5,9^.

The high incidence of thyroid nodules remains a subject of ongoing investigation. A prevailing hypothesis attributes the increased detection rate of thyroid nodules to the widespread adoption of advanced diagnostic technologies in recent years, suggesting that this surge in prevalence may be partly an artifact of enhanced detection capabilities^1^. However, attention is also being directed towards various non-congenital behavioral or environmental factors associated with the development of thyroid nodules. These include smoking, obesity, and iodine deficiency^10–12^. Despite numerous studies investigating various risk factors for thyroid nodules, there is a lack of in-depth research on the impact of long-term Bluetooth headset usage on the risk of developing thyroid nodules. This study seeks to fill this gap, providing a scientific basis for future epidemiological research and public health interventions^13,14^. Furthermore, the proliferation of mobile devices, particularly smartphones that rely on electromagnetic field (EMF) technology, has raised concerns about their potential non-ionizing radiation (NIR) effects on human health^15,16^. The thyroid gland is among the organs most susceptible to such influences^17^. Factors like the proximity of mobile phones to the thyroid gland during use and the gland's inherent sensitivity to radiation are noteworthy. While there is a consensus on the thyroid's sensitivity to ionizing radiation (e.g., radiotherapy, nuclear radiation)^18,19^, emerging evidence suggests that NIR, such as EMF and radiofrequency radiation (RFR) emitted by mobile phones, should not be overlooked in its potential impact on thyroid function, including elevations in Thyroid Stimulating Hormone (TSH) levels and disruption of the pituitary-thyroid axis^3,20^. Similarly, Bluetooth devices, which utilize short-wave transmissions at high speeds for communication and are commonly used in daily life, theoretically could have adverse effects on the thyroid gland, potentially influencing the development of thyroid nodules.

Shapley additive explanations (SHAP) represent an innovative method for interpreting machine learning models, integrating the concept of Shapley values from classical game theory to provide profound insights into model predictions^21^. Shapley values are used to measure the marginal contribution of each feature to the model output under different combinations of features, thereby ensuring a fair distribution of feature importance. The SHAP method calculates the average marginal contribution of each feature across all possible combinations, making the evaluation of feature contributions consistent and interpretable^22^. This method meticulously assesses the contributions of individual features within the model, enhancing the transparency and interpretability of otherwise complex and obscure machine learning constructs. SHAP's capabilities extend beyond elucidating the logic underpinning individual predictions; it offers a global perspective, thereby facilitating a comprehensive understanding of the model's operational dynamics at a macro level. This extensive explanatory power renders SHAP an invaluable and practical tool, particularly in applications where interpretability is paramount^23^.

Leveraging the potent explanatory capabilities of the SHAP method, this study endeavors to investigate the potential correlation between the usage of Bluetooth headsets and the incidence of thyroid nodules. We conducted a detailed analysis of variables such as the type of Bluetooth headset device, duration of use, and usage contexts, aiming to unveil the possible impacts of these ubiquitous technologies on thyroid health. By employing SHAP for the precise quantification and interpretation of influencing factors, this research aims to provide a scientific foundation for public health policymakers and practical health guidelines for the general public in using these technological products. Ultimately, this study aspires to enrich the understanding of the interplay between modern technology and human health.

## Result

### Sample characteristics

In this study, a total of 1000 questionnaires were distributed, from which 600 valid questionnaires were retrieved. These 600 valid questionnaires formed our complete dataset, utilized for descriptive statistical analysis as well as for assessing the reliability and validity of the questionnaire, thereby ensuring the accuracy and reliability of its design. Within the full dataset, respondents who utilized Bluetooth headsets comprised the "Bluetooth Headset User Dataset", which included a sample of 393 cases. This subset was employed not only for descriptive statistics and correlation analysis but also for t-SNE visualization and Propensity Score Matching (PSM) processing. The dataset subsequent to PSM processing, termed the "post-PSM dataset", encompassed a sample of 96 cases and was utilized for further descriptive statistics, modeling, and SHAP analysis.

### Descriptive statistics

Descriptive statistical analyses were conducted on three distinct datasets: the comprehensive dataset comprising 600 questionnaires, a subset dedicated to Bluetooth headset users with 393 individuals, and a dataset refined through Propensity Score Matching (PSM), including 96 questionnaires, aiming to assess the potential link between Bluetooth headset utilization and thyroid nodule prevalence. The findings indicated a considerable usage rate of 65.5% for Bluetooth headsets within the complete dataset, underscoring widespread use among participants. The incidence of thyroid nodules reported by Bluetooth headset users was consistent at 12.2% across both the full dataset and the user-specific subset. The PSM-adjusted dataset evenly balanced samples with and without thyroid nodules, each accounting for 50%, providing a balanced basis for in-depth analysis. Minor differences in average age across the datasets mirror the sample's age distribution. Additionally, listening to music emerged as the primary scenario for Bluetooth headset usage, predominantly employing in-ear types, showcasing uniform usage habits across the datasets (Table 1) .

**Table 1:** Descriptive statistics of the dataset.

	Full dataset	Bluetooth headset user dataset	Post-psm dataset
N	600	393	96
Age [mean (SD)]	31.43 (8.86)	30.50 (7.66)	30.8 (6.42)
Whether to use bluetooth headsets (%)			
Not use	207 (34.5)	0 (0)	0 (0 )
Use	393 (65.5)	393 (100)	96 (100)
Thyroid nodules (%)			
(Skip)	119 (19.8)	0 (0)	0 (0 )
No	408 (68.0)	345 (87.8)	48 (50)
Yes	73 (12.2)	48 (12.2)	48 (50)
Wearing style (%)			
(Skip)	207 (34.5)	0 (0)	0 (0)
In-ear style	297 (49.5)	297 (75.6)	62 (64.6)
Neckband style	39 (6.5)	39 (9.9)	14 (14.6)
Overhead style (earmuff style)	57 (9.5)	57 (14.5)	20 (20.8)
Usage scenarios (%)			
(Skip)	207 (34.5)	0 (0)	0 (0)
Listening to music	298 (50.0)	298 (75.8)	75 (78.1)
Navigation	18 (3.0)	18 (4.5)	5 (5.2)
Playing games	31 (5.2)	31 (7.8)	8 (8.3)
Work	46 (7.6)	46 (11.7)	8 (8.3)
Daily usage time (%)			
(Skip)	207 (34.5)	0 (0)	0 (0)
1 to 3 h	238 (39.6)	238 (60.6)	65 (67.7)
3 to 5 h	49 (8.1)	49 (12.5)	10 (10.4)
5 to 8 h	11 (1.8)	11(2.8)	4 (3.1)
8 to 12 h	3 (0.5)	3(0.8)	0 (0)
Less than 1 h	90 (15.0)	90(22.9)	18 (18.8 )
Wear continuously	2 ( 0.3)	2(0.5)	0 (0)
Commonly linked devices (%)			
(Skip)	207 (34.5)	0 (0)	0 (0)
Connect to computer	17 (2.8)	17 (4.3 )	6 (6.3)
Connect to mobile phone	376 (62.7)	376 (95.7)	90 (93.8)

Our study concentrates on exploring the relationship between Bluetooth headset usage habits and the occurrence of thyroid nodules. During the phase of data collection, we indeed took into account the variable concerning the operating system types of connected devices, such as Android and iOS. However, further examination indicated that this selection, although partially reflective of personal preferences, is substantially influenced by various objective factors, including but not limited to socioeconomic status and the disparity in pricing between most Android and iOS devices. These considerations exceed the purview and manageable parameters of our investigation, leading us to deduce that incorporating these variables into our final analysis could unnecessarily complicate the study and introduce potential biases. Moreover, it became apparent that any determinations regarding the system types of connected devices could significantly affect the commercial market and profoundly alter consumer purchasing behaviors and psychological states. In light of this, introducing variables potentially affected by wide-ranging external factors and making broad statements based on such would be irresponsible.

Hence, to uphold the integrity of our findings and demonstrate responsibility towards the public, we opted to exclude the analysis concerning the types of connected devices from this preliminary investigation. This deliberate choice is intended to ensure the precision and objectivity of our research outcomes, emphasizing the central question of our study: the potential linkage between Bluetooth headset usage habits and the prevalence of thyroid nodules.

### Correlation analysis

**Figure 1:**
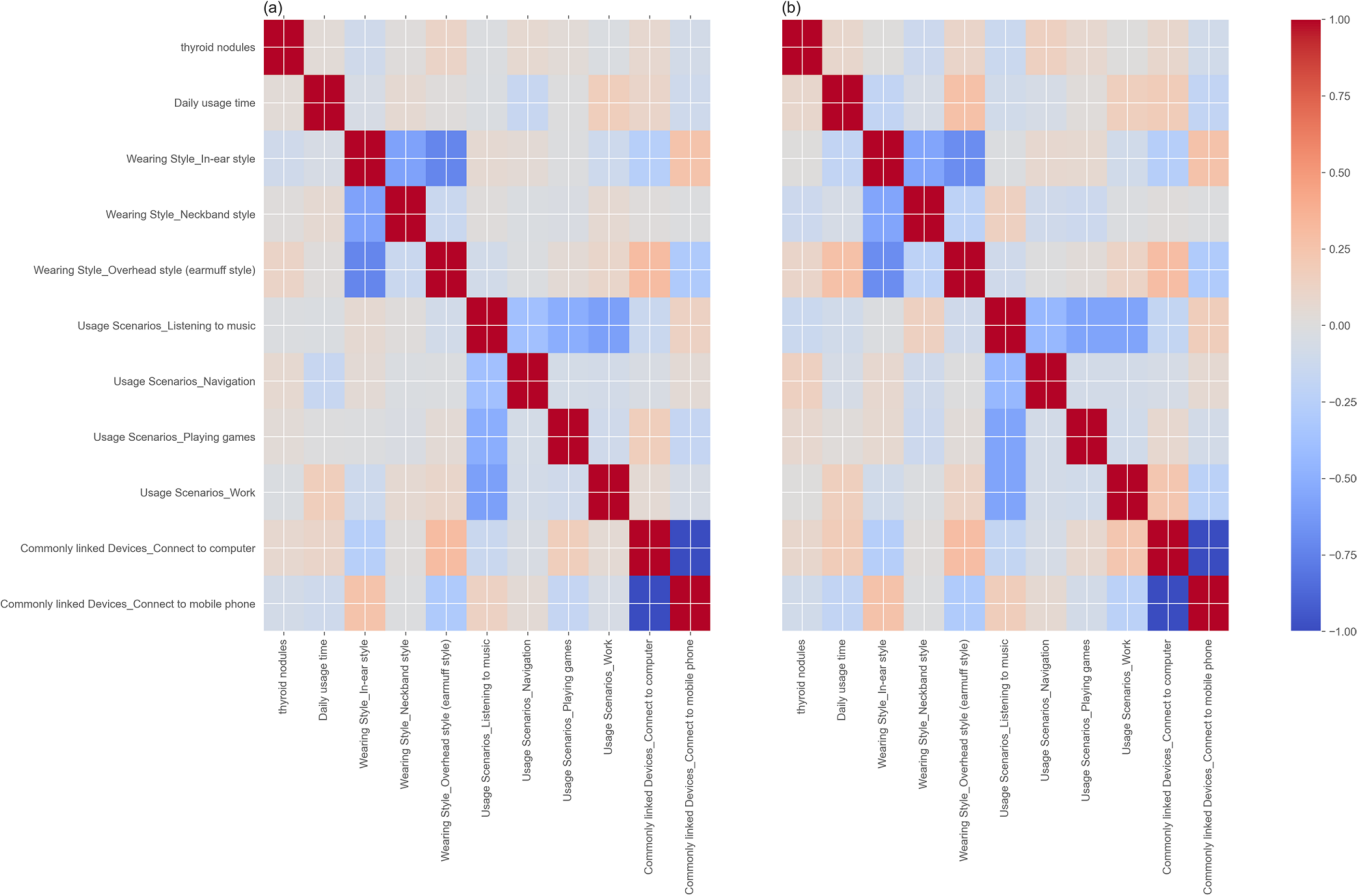
Correlation matrices of variables pre-PSM and post-PSM. (**a**) The correlation matrix before PSM, illustrating the initial associations between variables. (**b**) The correlation matrix after PSM.

A Spearman’s rank correlation analysis was conducted to decipher the relationships among categorical variables within our Bluetooth headset user dataset, as well as the post-PSM dataset (Fig. 1). The analysis yielded that current thyroid nodule status did not manifest significant correlation coefficients with other variables, suggesting that a more intricate and nuanced analysis would be required to unearth the underlying associations. Furthermore, the comparison of correlation matrices before and after PSM indicated that the matching process did not substantially alter the pre-existing trends in the data. Instead, it served to accentuate certain correlations which were already discernible, affirming the pre-match data's integrity and the matching technique's efficacy in enhancing the visibility of extant relationships.

### Thyroid nodule risk prediction modeling

**Figure 2:**
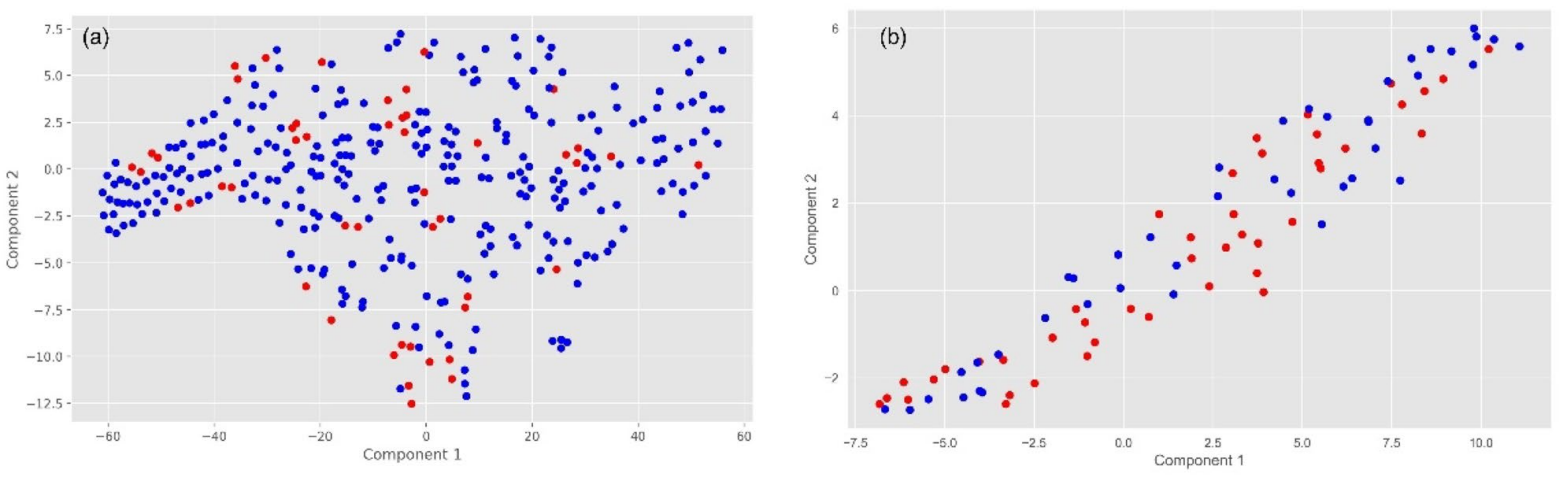
(**a**) Data distribution prior to propensity score matching. (**b**) Data distribution following propensity score matching. Each point maps a sample from high-dimensional space to two dimensions. Blue points indicate samples without thyroid nodules, and red points indicate those with nodules. Figure (**a**) shows data before propensity score matching (PSM), while (**b**) shows post-PSM data, illustrating how PSM adjusts covariate balance between groups and reduces bias. This helps compare distributions and analyze the PSM effect on sample structure.

**Figure 3:**
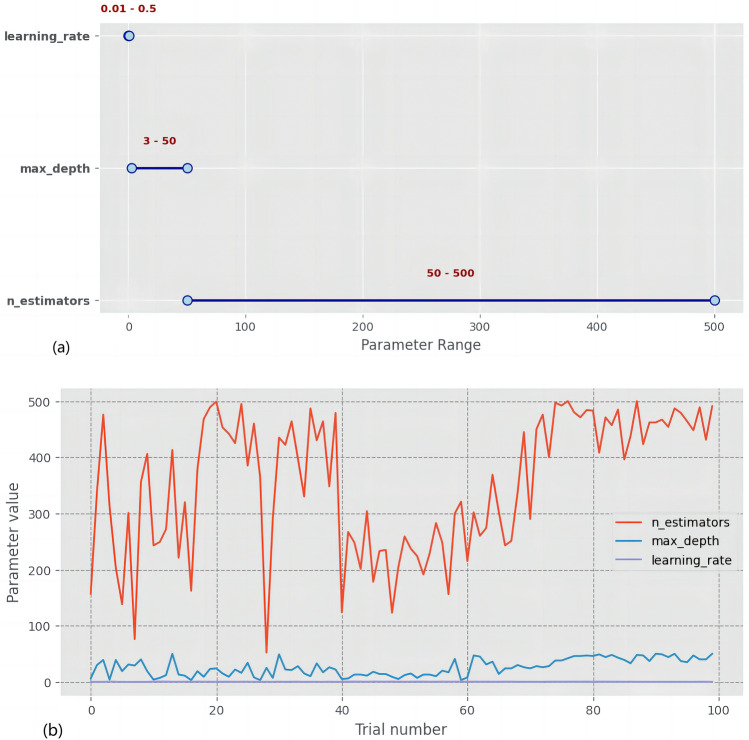
Optuna's hyperparameter search space (**a**) and the hyperparameter selection process (**b**).

**Figure 4:**
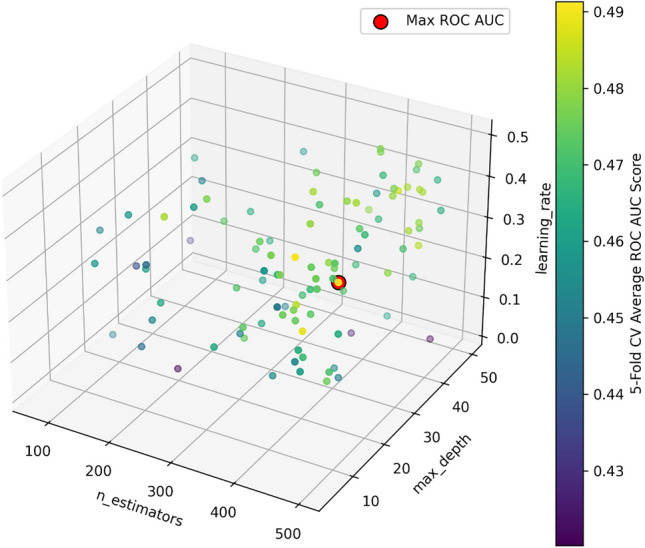
The average ROC-AUC values obtained from various hyperparameter combinations. The red circle indicates the coordinates of the hyperparameters at which the model achieves its maximum average ROC-AUC value.

In this research, to equalize the differences in outcome variables, we employed the Propensity Score Matching (PSM) method within the Bluetooth Headset User Dataset. We matched a control group of 48 samples from respondents who did not report thyroid nodules, creating a post-PSM dataset. The t-Distributed Stochastic Neighbor Embedding (t-SNE) method was used to visualize the effectiveness of the matching, which demonstrated a more uniform distribution of data post-matching compared to pre-matching (Fig. 2). This reduced potential selection bias, enhanced the representativeness of the data, and improved the validity of statistical inferences, facilitating the identification of potential associative patterns within the dataset during the modeling process. Building upon this, we employed the XGBOOST model, utilizing Optuna for automatic hyperparameter optimization to ensure the model's stability and predictive capacity. The hyperparameter search space utilized by Optuna is depicted in Fig. 3a. During Optuna's optimization process, each set of hyperparameters is subjected to a fivefold cross-validation, with the selection process for hyperparameters across all training iterations illustrated in Fig. 3b. The average ROC-AUC value from the cross-validation serves as the performance metric for the model, and the average ROC-AUC values obtained from different hyperparameter combinations are shown in Fig. 4. The point encircled in red represents the optimal hyperparameters, corresponding to the coordinates where the model achieved the highest average ROC-AUC value. Additionally, considering the logical independence of features, we excluded computer and mobile phone system options during modeling, as these are non-independent features highly dependent on commonly linked devices.

### ROC-AUC value

**Figure 5:**
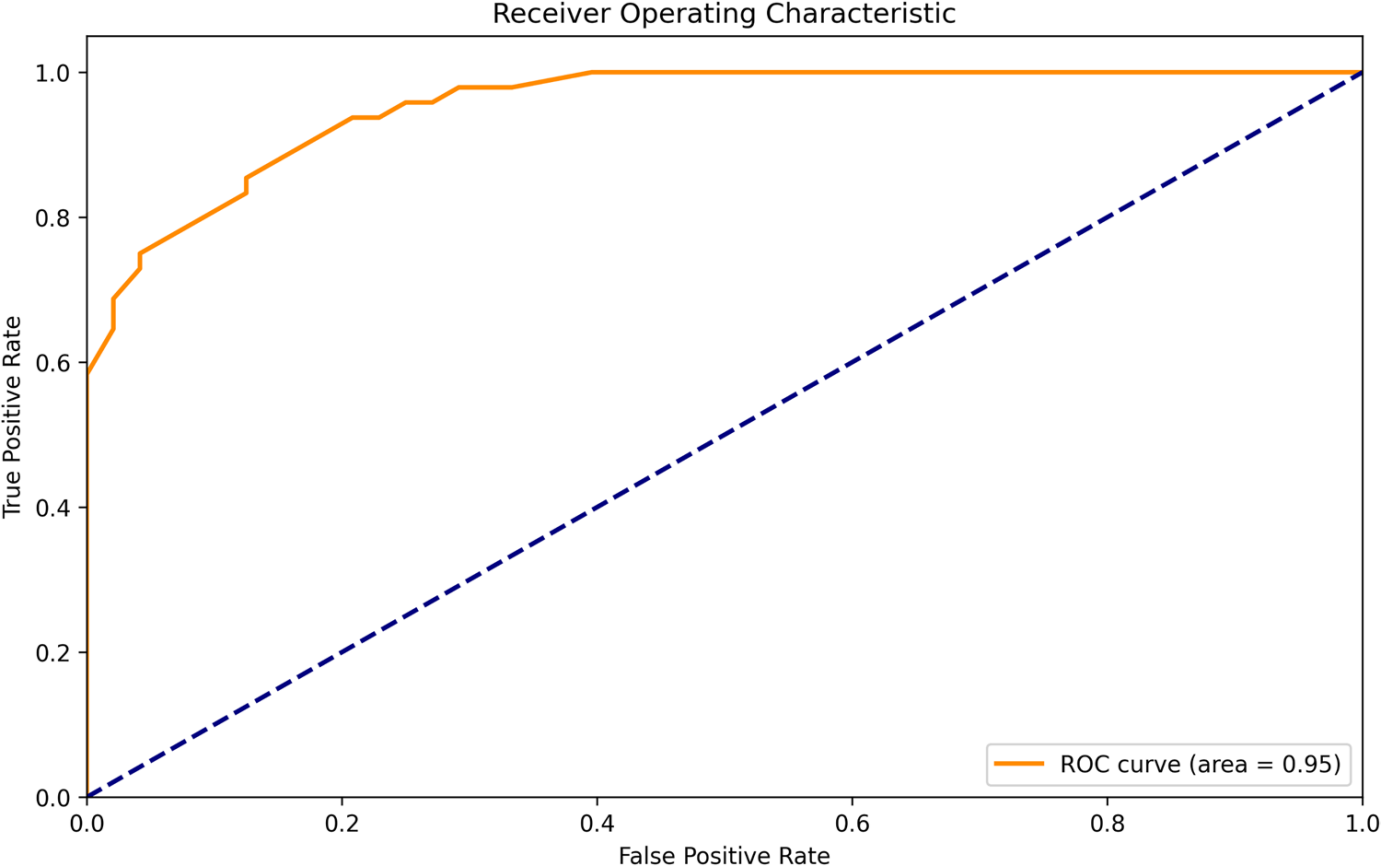
ROC curve of the XGBOOST model.

**Figure 6:**
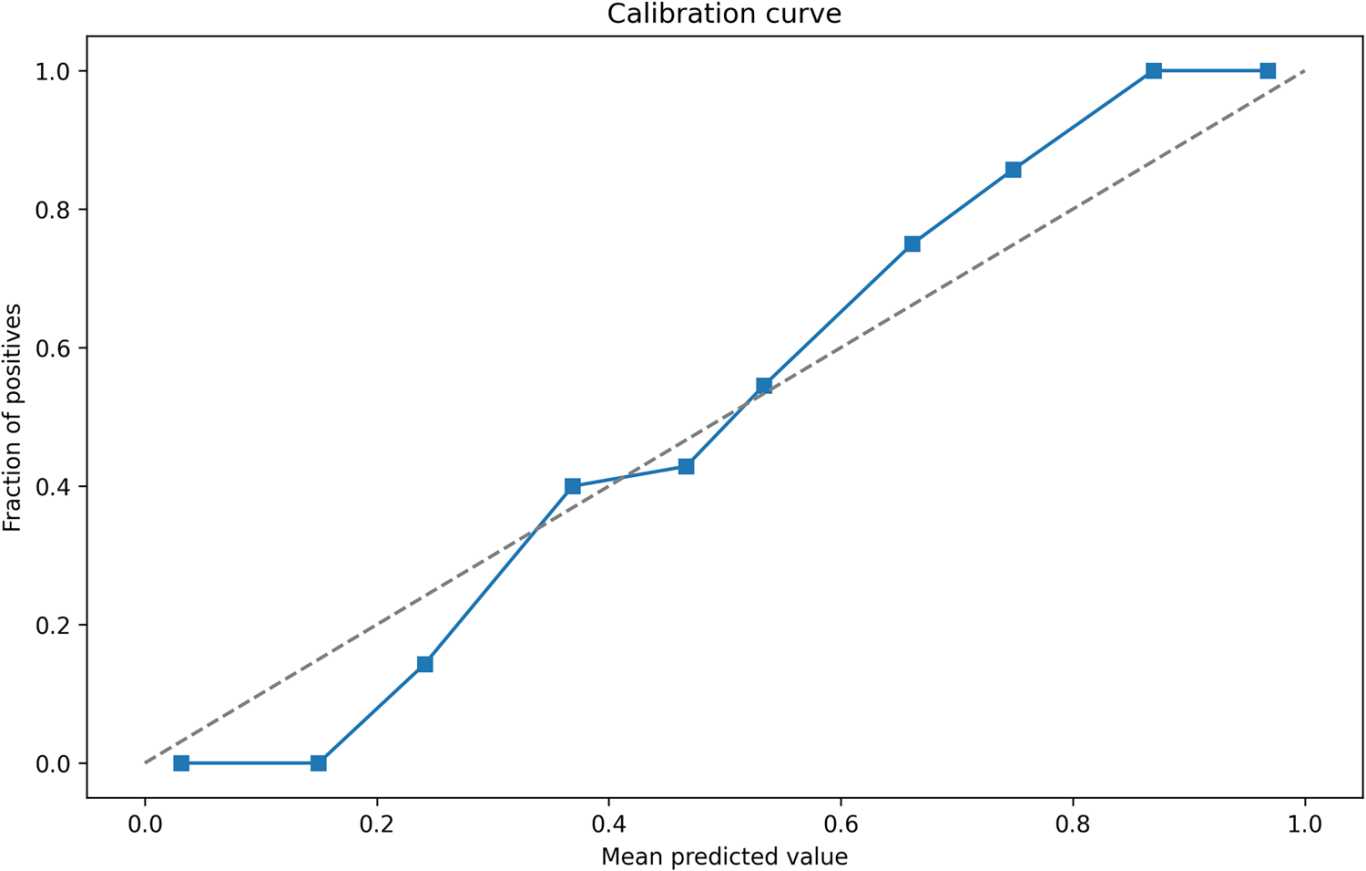
Calibration curve of the XGBOOST model.

The evaluation of the model's Receiver Operating Characteristic (ROC) curve and calibration curve demonstrated high precision and reliability in predicting the risk of thyroid nodules. The ROC curve's horizontal axis, representing the False Positive Rate (FPR), quantifies the proportion of actual negative cases incorrectly predicted as positive, reflecting the model's level of false alarms when differentiating between healthy states and thyroid nodule conditions. The vertical axis, indicating the True Positive Rate (TPR) or sensitivity, measures the proportion of actual positive cases correctly identified, directly assessing the model's capability to detect thyroid nodule samples. The Area Under the ROC Curve (AUC) valued at 0.95, signifies the model's heightened sensitivity in distinguishing between positive and negative thyroid nodule samples, suggesting an exemplary overall classification performance (Figs. 5). The calibration curve serves as an essential tool for evaluating the accuracy of the model's predicted probabilities. In the calibration curve, the horizontal axis represents the Predicted Probability, while the vertical axis denotes the Actual Probability of events occurring. An ideal calibration curve is a straight line originating from the zero point with a slope of 1, indicating a perfect alignment between the model's predicted probabilities and the actual event probabilities. In our research, the calibration curve closely approximates this ideal line, indicating a high consistency between the model's predicted risks for thyroid nodules and the actual observed risks, thereby affirming the model's predictive accuracy's high degree of reliability (Figs. 6).

### SHAP analysis of thyroid nodule risk factors

**Figure 7:**
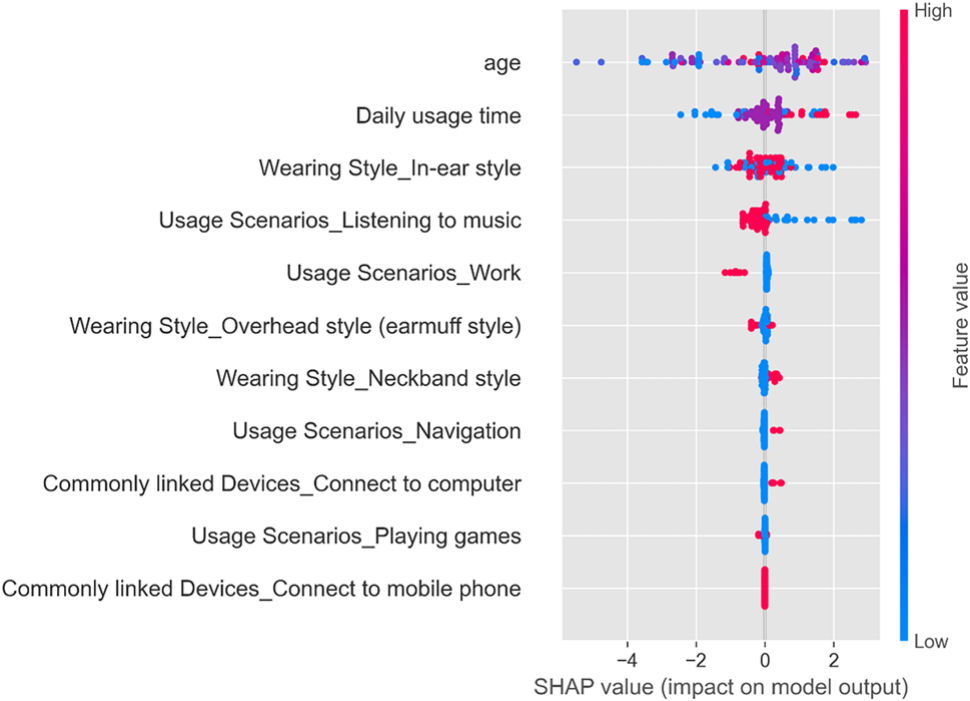
SHAP summary plot.

**Figure 8:**
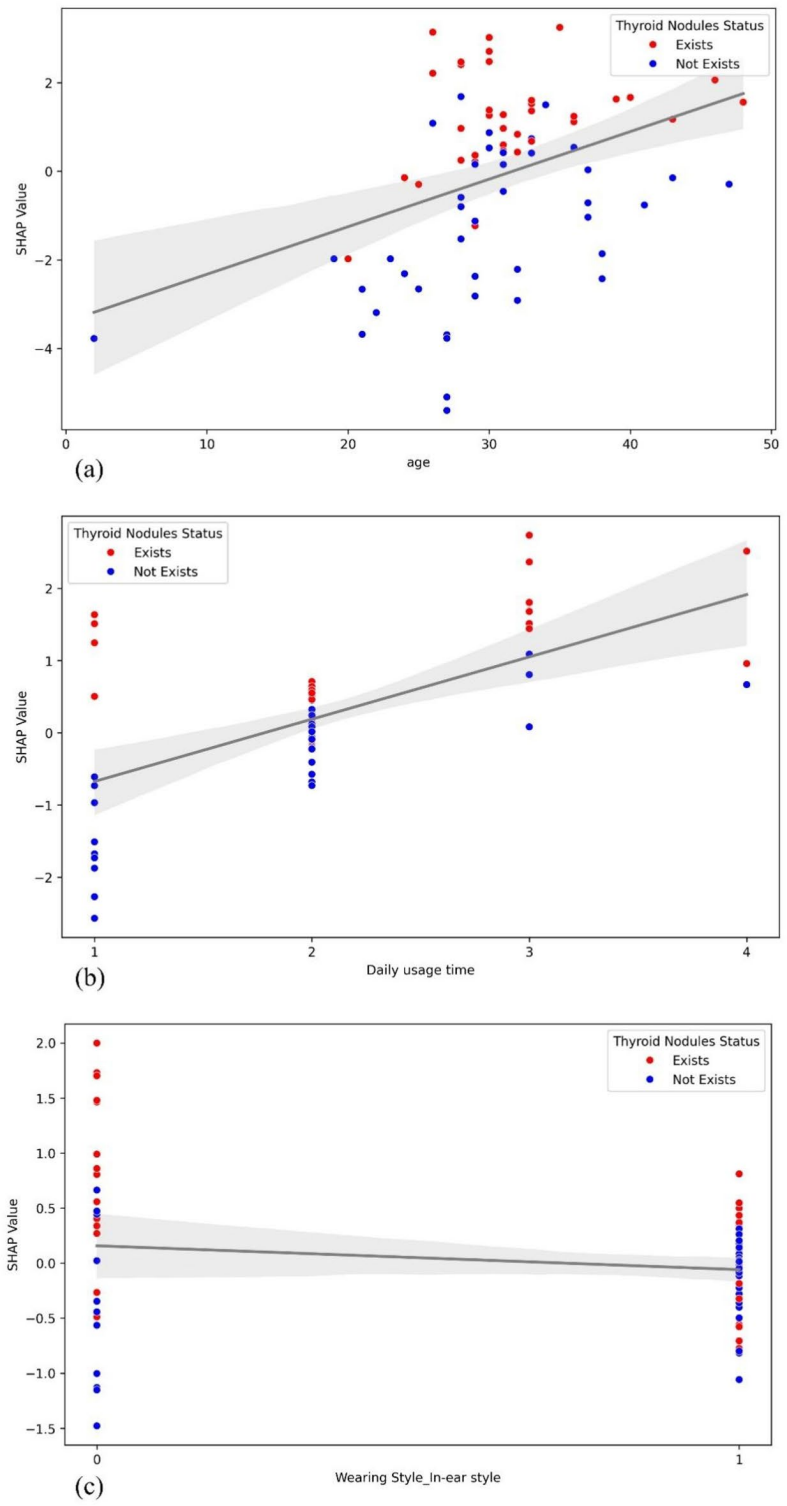
Scatter plots of SHAP dependencies for the top three factors in the SHAP summary plot. (**a**) SHAP values vs. age with trend line. (**b**) SHAP values vs. daily usage time with trend line. (**c**) SHAP values vs. wearing style in-ear style with trend line.

In the SHAP summary plot (Fig. 7), the vertical ordering of features is based upon the magnitude of their average absolute SHAP values, revealing the relative importance of each feature in the predictive model output. Each horizontally distributed point represents an individual observation, with its position on the horizontal axis indicating the marginal contribution of the feature value to the probability of predicting the risk of thyroid nodules. Positive SHAP values (points to the right of the zero line) indicate that higher values of the feature increase the likelihood of the model predicting the occurrence of thyroid nodules; conversely, negative SHAP values (points to the left of the zero line) suggest that lower values of the feature are associated with a reduced probability of nodules. The depth of color (typically red for high values and blue for low values) reflects the actual magnitude of the feature value, visually conveying the specific impact of the feature's value on the model output. To further highlight this impact, the SHAP summary plot's top three most important features were graphically represented in relation to the model's output target through dependence plots, which showed a clear positive influence of the age and daily usage time variables on SHAP values, as illustrated in the scatter plots with trend lines (Fig. 8).

The trend observed in the age feature corresponds with the broadly accepted medical consensus that an increase in age is a significant risk factor for the development of thyroid nodules. This relationship is quantitatively substantiated in our analysis, affirming the role of age as a determinant. Regarding the "Daily usage time" feature, observed positive SHAP values indicate a correlation between extended use of Bluetooth headsets and an increased risk of thyroid nodules within our dataset. Variations in point color further refine this interpretation—the deep red points correspond to longer usage durations, suggesting a higher risk association, while deep blue points imply shorter durations and a lower risk correlation. This color coding allows for a richer interpretation from each data point.

## Discussion

Bluetooth technology is a form of wireless communication developed by the Swedish telecommunications equipment manufacturer Ericsson in 1994. It facilitates short-range wireless interactions between electronic devices. In 1998, Ericsson evolved Bluetooth into a consortium comprising multiple computer and electronic companies, subsequently gaining widespread popularity in consumer devices^24^. Over time, its application has expanded considerably, making it a globally recognized standard for wireless communication. The core of Bluetooth technology is based on the IEEE 802.15 protocol, which employs low-power radio waves in the 2.400 GHz to 2.480 GHz ISM (Industrial, Scientific, and Medical) frequency band, one of the several bands designated by international agreements for industrial, scientific, and medical device usage^25,26^. Bluetooth achieves short-range, efficient wireless communication between electronic devices by utilizing frequency-hopping spread spectrum technology and standardized security protocols.

Bluetooth technology, like the wireless technology used in mobile phones and other everyday communication devices, generates NIR. Compared to mobile phone communication bands ranging from 3 to 300 GHz, the short-wavelength, ultra-high-frequency radio waves used by Bluetooth are generally considered to produce weaker radiation. Although NIR lacks the energy to dislodge electrons from atoms and directly cause chemical bond breaks or induce cancer, this does not imply that it is entirely harmless. In vitro experiments by Duan et al. (2015) on mouse spermatogonial cell lines found that exposure to 50 Hz and 1800 MHz ELF-EMF led to a slight increase in DNA strand breaks, with electromagnetic frequency exposure alone having no effect. Advanced alkaline comet assays further revealed that RF-EMF significantly increased oxidative DNA damage, whereas ELF-EMF exposure did not have a similar effect ^27^. Hou et al. (2015) investigated the impact of 1800 MHz frequency radiation on embryonic fibroblasts. Results showed increased levels of reactive oxygen stress and apoptosis (secondary necrosis) in cells, as well as a slight increase in DNA strand breaks after 1, 4, and 8 h of RFR exposure ^28^. Animal studies revealed that rats exposed to 50 Hz EMF exhibited an increased number of A-type degranulated mast cells in the thyroid, potentially due to mediators released by these cells. Further research found a significant increase in histamine-containing mast cells and neuropeptide Y (NPY) nerve fibers in the thyroid of rats exposed to extremely low-frequency electromagnetic fields (ELF-EMF), potentially enhancing thyroid microvascular blood flow and permeability^29,30^. In human studies, a 2004 cross-sectional study by Bergamaschi et al. on 2598 employees investigated the impact of mobile phone usage frequency on thyroid function. The study found that the prevalence of low TSH values was 9.9% in the employee group using mobile phones for more than 33 h per month, compared to 6% in the group using less than 19 h per month. This suggests that mobile phone radiation may directly affect thyroid tissue or indirectly impact thyroid function and hormone production by interfering with the pituitary-thyroid axis, leading to increased or decreased serum TSH values and thyroid dysfunction following RFR exposure^31^. Other population studies have shown that prolonged, high-intensity NIR radiation significantly affects TSH and T4 levels, decreases T3 levels, and may cause thyroid dysfunction, negatively impacting the hypothalamic-pituitary-thyroid axis ^2,32^. These studies collectively indicate that NIR has significant biological effects, from DNA damage and increased oxidative stress to thyroid dysfunction, as evidenced across various levels from in vitro cellular experiments and animal studies to human research, highlighting the potential health risks of NIR. Despite the lack of sufficient high-level epidemiological evidence to directly impact medical decision-making, existing findings have still attracted some attention. Previous studies have aided in understanding the biological effects of non-ionizing radiation (NIR), but research specifically addressing the impact of Bluetooth headsets, a particular source of radiation, in populations remains very limited. This study aims to bridge this gap by exploring the potential association between the frequency of Bluetooth headset use and the risk of thyroid nodules.

The analysis of age factors in this study revealed a significant trend: susceptibility to thyroid nodules increases with age. This phenomenon may be partly attributed to the gradual decline in the body's self-repair capabilities and cellular regeneration mechanisms with aging, leading to cumulative effects on cell damage and increased health risks^33,34^. Additionally, the analysis of daily headphone usage time (daily_usage_time) indicated a critical cumulative effect: prolonged daily use of NIR sources like Bluetooth headphones may increase the risk of thyroid nodules. This cumulative effect reflects that even though NIR is not sufficient to directly cause cellular ionization, its long-term effects could still exert subtle negative impacts on cellular functions. Notably, the thyroid is an organ highly sensitive to radiation, and prolonged NIR exposure may lead to damage in this organ. This hypothesis is supported by SHAP analysis in individuals who wear Bluetooth headphones for extended periods, suggesting that health guidance in the use of everyday electronic devices should particularly focus on their potential long-term impacts on the thyroid. This consideration is crucial for the development of preventive measures and public health recommendations, necessitating an increased awareness of the potential health impacts of electronic device usage while promoting their use.

The widespread use of Bluetooth headphones, particularly as a daily accessory for prolonged periods among younger populations, has raised concerns about their long-term health effects. Given the thyroid's high sensitivity to radiation, long-term exposure to NIR, especially from Bluetooth headphones worn close to the neck, might intensify the impact on this organ. Our study's analysis using SHAP values indicates a correlation between daily headphone usage time and an increased risk of thyroid nodules, reinforcing the hypothesis of a cumulative effect of NIR on the thyroid. Therefore, it is imperative to consider thyroid health as a critical factor in the assessment of the long-term impacts of Bluetooth headphone usage in the formulation of future public health policies and health guidelines. We recommend conducting more in-depth research to precisely understand the specific effects of long-term Bluetooth headphone usage on the risk of thyroid nodules and to develop targeted preventive measures to mitigate potential health risks.

This study is subject to several limitations. Initially, while the SHAP values offer a quantified measure of the impact on predictive model features, these metrics do not inherently imply causality. The increase in age and the extended duration of headphone usage may merely serve as indicators of heightened risk for thyroid nodules, with their variations potentially linked to other variables not observed in this study. Furthermore, considering that the duration of headphone usage is based on participant self-reporting, a certain degree of subjectivity is inherent, potentially introducing bias. Thus, future research should employ more precise tracking technologies for usage duration to enhance the objectivity and reliability of the data. Additionally, the data for this study were sourced from surveys of mobile smart device users, a method that tends to attract a younger demographic, potentially leading to sampling bias. Younger individuals might be more inclined to prolonged Bluetooth headphone usage, which could affect the assessment and interpretation of risk factors for thyroid nodules. Consequently, our findings may not be entirely applicable to a broader population. Future research should incorporate a more diversified sampling strategy that includes individuals across a broader spectrum of ages and socio-economic backgrounds to more comprehensively evaluate the association between Bluetooth headphone usage and the risk of thyroid nodules.

## Conclusion

This investigation delved into the correlation between thyroid nodules and the use of Bluetooth headphones, elucidating the potential implications of NIR in daily life, particularly its impact on thyroid health. The findings suggest that besides age, a known factor, prolonged and frequent use of Bluetooth headphones may be associated with an increased risk of developing thyroid nodules, potentially linked to the cumulative effects of NIR emitted by the headphones on the thyroid gland. Moreover, this study underscores the necessity of giving special attention to thyroid health while assessing the health risks of modern wireless technologies. Although this research offers valuable insights, its limitations preclude direct inferences of causality. Future research should further investigate the specific relationship between long-term use of Bluetooth headphones and the risk of thyroid nodules, and develop more precise preventive measures to mitigate the potential health risks posed by NIR. Additionally, the findings of this study provide crucial guidance for public health policymakers, aiding in the promotion of modern technology while simultaneously enhancing public awareness and prevention of its potential health impacts.

## Method

### Data source

The data for this study was sourced from the sample service of WenJuanXing, a professional online platform dedicated to surveys, exams, assessments, and voting. WenJuanXing is committed to providing users with robust and user-friendly services for the design of online questionnaires, data collection, custom reporting, and the analysis of survey results. The questionnaire used in this research included questions on age, presence of thyroid nodules, use of Bluetooth headsets, wearing style of Bluetooth headsets (In-ear style, Neckband style, Earmuff style), primary usage scenarios of Bluetooth headsets (Listening to music, Navigation, Playing games, Work), daily usage duration (Less than 1 h, 1 to 3 h, 3 to 5 h, 5 to 8 h, 8 to 12 h, Wear continuously), commonly connected devices for the Bluetooth headset (Computer, Mobile phone), type of computer operating system (Windows, Mac OS, Linux, Chrome OS, Other, Unsure), and type of mobile phone operating system (Android, iOS, Symbian/BlackBerry/Windows Phone, Other, Unsure) (Table 2). Given the diversity of the target population for social surveys, the aforementioned questions and options were described in a more understandable manner in the questionnaire. This project aimed to collect data from 1,000 participants, with WenJuanXing randomly distributing the questionnaire to its users. Participation in this survey was voluntary, and users had the option to decline or disregard the research invitation. This study has been reviewed by the Ethics Review Committee of Dongtai City Traditional Chinese Medicine Hospital and has received a written exemption from ethical review. All methods were performed in accordance with the relevant guidelines and regulations.

**Table 2:** Questions and options in the questionnaire.

Section	Question	Options
Basic information	Q1: what is your age?	–
	Q2: do you have thyroid nodules?	◎ Yes ◎ No ◎ Unsure
Usage of bluetooth headsets	Q3: do you use bluetooth headsets?	◎ I often use it ◎ I never use it ◎ Other–
	Q4: what type of wearing style do you typically use for bluetooth headsets?	◎ In-ear ◎ Neckband ◎ Over-ear (earmuff) ◎ Other–
	Q5: in which scenarios do you primarily use bluetooth headsets?	◎ Listening to music ◎ Navigation ◎ Playing games ◎ Work ◎ Other_
	Q6: what is your daily duration of bluetooth headset usage?	◎ Less than 1 h ◎ 1 to 3 h ◎ 3 to 5 h ◎ 5 to 8 h ◎ 8 to 12 h ◎ Continuous wearing ◎ Other_
	Q7: which devices do you commonly connect to your bluetooth headset?	◎ Computer ◎ Mobile device
Technological preferences	Q8: what operating system does your computer use?	◎ Windows ◎ Mac os ◎ Linux ◎ Chrome os ◎ Other_ ◎ Unsure
	Q9: what is the operating system of your mobile phone?	◎ Android ◎ Ios ◎ Symbian ◎ Blackberry/windows/phone ◎ Other_ ◎ Unsure

### Data preprocessing

In the data preprocessing phase of this study, we implemented a series of stringent measures to ensure the quality and consistency of the data. Initially, automated screening procedures were employed to identify and exclude invalid responses, including incomplete submissions, untimely submissions, or forceful exits during the questionnaire completion. This step was crucial to maintain the integrity and reliability of the dataset. Given the high heterogeneity inherent in social survey data, a meticulous review of the questionnaire responses was conducted. Any survey that did not adhere to the specified format, such as those randomly filled or containing gibberish, was eliminated to ensure the cleanliness of the dataset and the accuracy of the analysis.

In addressing missing values, a phased strategy was adopted. Variables with more than 40% missing values were completely excluded from the analysis, considering that a high proportion of missing data could severely affect the accuracy of the results. However, this rule did not apply to special response logic, such as questions skipped based on certain preceding options. For variables with less than 40% missing values, different imputation strategies were employed. For categorical variables, the mode was used for imputation, while for continuous variables, mean imputation was utilized. This approach aimed to preserve the statistical characteristics of the data and minimize the impact of missing values on the analysis results. Furthermore, for unordered multicategorical variables, One-Hot Encoding was applied, whereas ordered multicategorical variables were encoded according to their ordinal levels. This treatment facilitated the precision and interpretability of subsequent model analyses. Additionally, for free-text responses elicited by the "Other" options within survey questions, we utilized regular expressions for keyword matching. This approach allowed us to categorize or infer the inclination of their responses based on the keywords identified within these open-ended answers.

### Dataset segmentation

**Figure 9:**
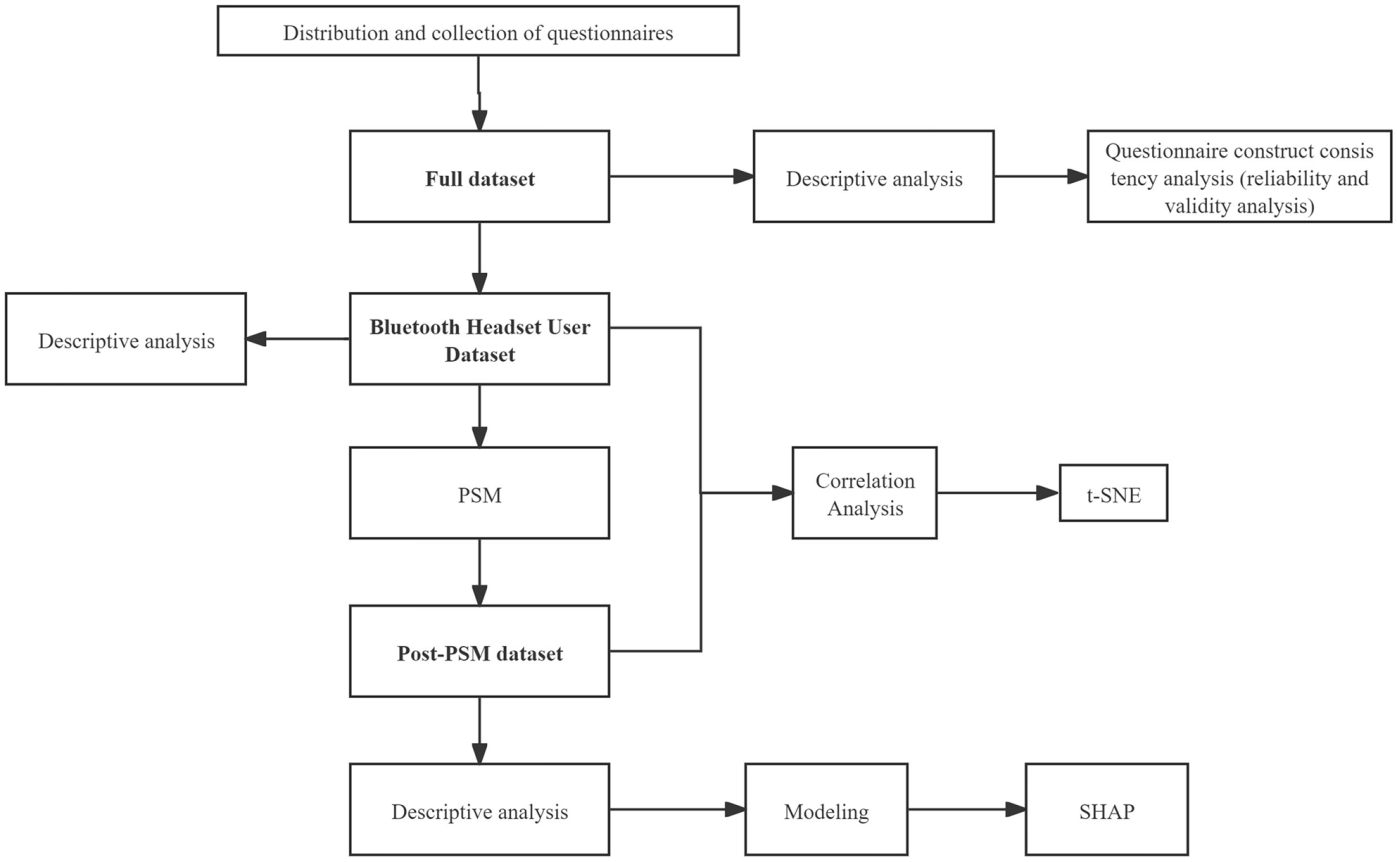
Research flow chart. *PSM* propensity score matching, *t-SNE*: t-distributed stochastic neighbor embedding, *SHAP* Shapley additive explanations.

The rationale behind our dataset segmentation is multifaceted. Firstly, the analysis of reliability and validity focuses on the scientificity and rationality of the questionnaire design concept, aiding in ensuring the quality of the collected data and the reliability of the questionnaire, consequently, the full dataset will be employed in this analysis step. Secondly, given that our questionnaire includes numerous questions related to Bluetooth headset usage habits, data from non-users of Bluetooth headsets were automatically marked as “skip” for these questions. These samples hold no analytical value for research into Bluetooth headset usage habits and their health impacts. Therefore, these respondents were excluded from further analysis related to Bluetooth headset use and habits. Lastly, this study focuses on investigating the potential correlation between the prevalence of thyroid nodules and the usage of Bluetooth headsets. The initial dataset revealed a significant imbalance between samples with and without thyroid nodules. To address this, the subsequent modeling and SHAP analysis utilized the post-PSM dataset for more accurate results. The segmentation and application of the dataset are illustrated in Fig. 9.

### Descriptive statistics

In this study, we conducted a comprehensive descriptive statistical analysis on the entire dataset. For continuous variables, their distribution characteristics were described using the mean ± standard deviation. For categorical variables, counts and percentages were employed for presentation. Furthermore, a correlation analysis was performed specifically on the dataset of Bluetooth headset users to uncover simple patterns of association between variables. This is because non-use of Bluetooth headsets implies that most questions in the questionnaire would be automatically skipped, making the significance of conducting a correlation analysis across the full dataset unclear from a logical standpoint.

### Applying PSM for data balancing

In this study, we adopted the propensity score matching (PSM) technique to balance the differences between the datasets. Modeling in extremely unbalanced data environments may increase the risk of model overfitting, where the model tends to identify the more numerous categories and overlook equally important minority categories. This not only obscures genuine and subtle patterns and relationships but also impairs the model's ability to capture key signals. By using PSM, we are able to directly counteract and alleviate the bias caused by data imbalance, providing the model with a more balanced and representative data foundation, thereby enhancing the predictive accuracy and reliability of the model. Further, the efficacy of the matching process was demonstrated through the utilization of t-SNE technology, a sophisticated non-linear dimensionality reduction technique that preserves the local structure of high-dimensional data by embedding it into a lower-dimensional space. By calculating the similarity between points in the high-dimensional space and representing these relationships in a lower-dimensional map, t-SNE enables a visual assessment of data homogeneity post-Propensity Score Matching (PSM). In this study, applying t-SNE facilitated the visualization of the balanced distribution of treatment and control groups, thereby substantiating the reduction of potential biases between groups. This methodological approach not only validates the effectiveness of our matching strategy but also lays a robust foundation for subsequent analyses of SHAP values, ensuring the dataset's homogeneity and the reliability of our inferential statistics. Through t-SNE, we were able to achieve our objective of demonstrating a significant improvement in the balance of key variables across groups, essential for accurate causal inference in SHAP value analysis.

### SHAP enhanced XGBOOST modeling for thyroid risk factors

In this study, we utilized a specific dataset and employed the SHAP method based on the XGBOOST model to thoroughly analyze the factors influencing the risk of thyroid nodules and their complex interrelationships. The SHAP method calculates the average marginal contribution of each feature across all possible combinations, ensuring a fair distribution of feature importance and making the evaluation of feature contributions consistent and interpretable. In our study, we used SHAP summary plots and dependence plots to present the SHAP values for each feature, revealing the key factors influencing the model's predictions. The SHAP summary plot ranks features by their mean absolute SHAP values, displaying the relative importance of each feature across all samples, while the dependence plots further elucidate the interactions between features and their impact on the model output. In this manner, SHAP not only enhances the transparency and interpretability of the model's predictions but also provides a comprehensive understanding of the model's operational dynamics from a global perspective. This extensive explanatory power makes SHAP an invaluable tool, particularly in applications where interpretability is crucial. Additionally, during the modeling process, we utilized Optuna for hyperparameter optimization, further enhancing the model's performance and ensuring the reliability of the SHAP analysis based on this model.

### Analytical tools and environments used

Python 3.11 served as the primary platform for data analysis and model development. The study integrated several libraries, including pandas for data manipulation, sklearn for machine learning modeling, matplotlib for data visualization, xgboost for efficient model training, Optuna for automated hyperparameter optimization, and SHAP for interpreting the model's predictions. All Python-related development and testing activities were conducted within DataSpell, an integrated development environment developed by JetBrains. Moreover, propensity score matching analysis was executed using R language version 4.3.2, with R Studio serving as the development environment. The utilization of these advanced tools ensured the efficiency and accuracy of the research's analytical processes.

### Statement on ethical review

Ethics Review Committee of Dongtai City Traditional Chinese Medicine Hospital waived need for approval and informed consent.

## Data Availability

The datasets generated during and analysed during the current study are available from the corresponding author on reasonable request.

## Abbreviations

NIR: Non-ionizing radiation
SHAP: Shapley additive explanations
XGBOOST: EXtreme gradient boosting
PSM: Propensity score matching
t-SNE: t-Distributed stochastic neighbor embedding
ROC: Receiver operating characteristic
AUC: Area under the curve
ELF-EMF: Extremely low frequency-electromagnetic fields
RF-EMF: Radiofrequency electromagnetic fields
NPY: Neuropeptide Y
TSH: Thyroid stimulating hormone
ISM: Industrial, scientific, and medical (frequency band)
IEEE: Institute of Electrical and Electronics Engineers
RFR: Radiofrequency radiation
